# Tumor-Induced Osteomalacia Localized by Systemic Venous Sampling and ^68^Ga-DOTATOC Positron Emission Tomography

**DOI:** 10.1210/jcemcr/luaf012

**Published:** 2025-01-24

**Authors:** Tomomi Nakao, Ken Takeshima, Shuhei Morita, Ichiro Yamauchi, Sho Koyasu, Taka-Aki Matsuoka

**Affiliations:** First Department of Internal Medicine, Wakayama Medical University, Wakayama City, Wakayama 641-8509, Japan; First Department of Internal Medicine, Wakayama Medical University, Wakayama City, Wakayama 641-8509, Japan; First Department of Internal Medicine, Wakayama Medical University, Wakayama City, Wakayama 641-8509, Japan; Department of Diabetes, Endocrinology and Nutrition, Kyoto University Graduate School of Medicine, Kyoto 606-8507, Japan; Department of Radiology, Kyoto University Hospital, Kyoto 606-8507, Japan; First Department of Internal Medicine, Wakayama Medical University, Wakayama City, Wakayama 641-8509, Japan

**Keywords:** tumor-induced osteomalacia, fibroblast growth factor 23, ^111^In-pentetreotide scintigraphy, systemic venous sampling, ^68^Ga-DOTATOC-PET/CT

## Abstract

Tumor-induced osteomalacia is characterized by hypophosphatemia and fragility fractures caused by fibroblast growth factor 23 (FGF23)-producing tumors. We report a case of tumor-induced osteomalacia in which the tumor location could be determined by gallium 68 (^68^Ga)-DOTATOC positron emission tomography (PET)/computed tomography (CT). A 74-year-old woman had recurrent fractures and bone pain. Blood tests showed hypophosphatemia and elevated serum alkaline phosphatase and FGF23 levels and CT and bone scintigraphy showed multiple bone fractures. Tumor-induced osteomalacia was therefore suspected. Indium 111 (^111^In)-pentetreotide scintigraphy showed focus of increased activity in the head, and CT and magnetic resonance images showed a mass-like lesion in the posterior ethmoidal sinus. However, in systemic venous sampling, serum FGF23 level was highest in the left common iliac vein. ^68^Ga-DOTATOC PET/CT clearly demonstrated focal uptake in the left anterior inferior iliac spine consistent with systemic venous sampling. Retrospectively analyzed, focal uptake in the head was considered to be a physiological uptake in the pituitary gland. The tumor was resected and the pathological diagnosis was phosphaturic mesenchymal tumor. A combination of systemic venous sampling and ^68^Ga-DOTATOC PET/CT was useful in detection of a small FGF23-producing tumor. Precise tumor localization in such cases requires careful interpretation of scintigraphy.

## Introduction

Tumor-induced osteomalacia (TIO) is a paraneoplastic syndrome caused by fibroblast growth factor 23 (FGF23)-producing tumors originating from soft tissue and bone; it is characterized by hypophosphatemia and fragility fractures [1, 2]. Patients with TIO are often undiagnosed for a long time, owing to nonspecific symptoms, such as bone pain and muscle weakness [3]. Also, FGF23-producing tumors are often too small to localize [4].

Somatostatin receptor (SSTR) scintigraphy is an imaging method for visualizing SSTR expressed in neuroendocrine tumors [5]. Among various forms of SSTR scintigraphy, only indium 111 (^111^In)-pentetreotide scintigraphy is currently covered by National Health Insurance in Japan for neuroendocrine tumors. However, the sensitivity is relatively low, and there should be careful consideration of physiological accumulation in normal organs [6, 7]. Systemic venous sampling (SVS) of FGF23 is also reportedly useful for localizing FGF23-producing tumors, but it is an invasive method [8]. A more accurate imaging method for detecting TIO is therefore desired. Gallium 68 (^68^Ga)-DOTATOC positron emission tomography (PET)/computed tomography (CT) has reported high sensitivity and specificity in the localization of TIO [4].

## Case Presentation

A 74-year-old woman had recurrent fractures and bone pain throughout the body. She never smoked or drank alcohol and had no abnormal eating behavior. She had chronic back pain from approximately 50 years old and reached menopause at 51 years old. Annual physical examination revealed high levels of serum alkaline phosphatase (ALP), but no additional work-up had been performed.

She had a fall at age 55 years and began to feel pain in her left foot. A nearby hospital diagnosed left third metatarsal fracture. One month later, follow-up radiography revealed additional fractures of the first, second, and fourth metatarsal bones. On retrospective review, blood tests from routine annual physical examination showed low serum phosphate levels of 1.8 mg/dL (0.58 mmol/L) (reference range, 2.7-4.6 mg/dL; 0.87-1.49 mmol/L). Osteoporosis was diagnosed when she was 58 years of age, and the patient started medication with alfacalcidol 1.0 µg/day and alendronate sodium hydrate 5 mg/day, aiming to prevent further fractures.

At 73 years old, the patient felt worsening of back pain, especially when carrying heavy luggage or turning in bed. She gradually developed bilateral leg and hip pain, so she visited our orthopedic department. Spine radiography showed degenerative scoliosis. CT imaging revealed a fifth transverse fracture of the lumbar spine, right femoral trochanteric fracture, and pelvic fractures involving the pubis and ischium. She was treated with romosozumab 210 mg/month, an anti-sclerostin monoclonal antibody, for osteoporosis with severely high risk of fractures. Blood chemistry tests showed low serum phosphate levels of 1.8 mg/dL (0.58 mmol/L) and elevated serum FGF23 levels of 226 pg/mL (reference range, < 30 pg/mL). TIO was therefore suspected, and the patient was referred to the First Department of Internal Medicine. On admission, she presented with lower back pain, neck pain, and right shoulder pain at rest. She could barely walk on her own feet, needing to hold onto walls or handrails for support. She could not stand on one leg at that time.

## Diagnostic Assessment

**Figure 1.**
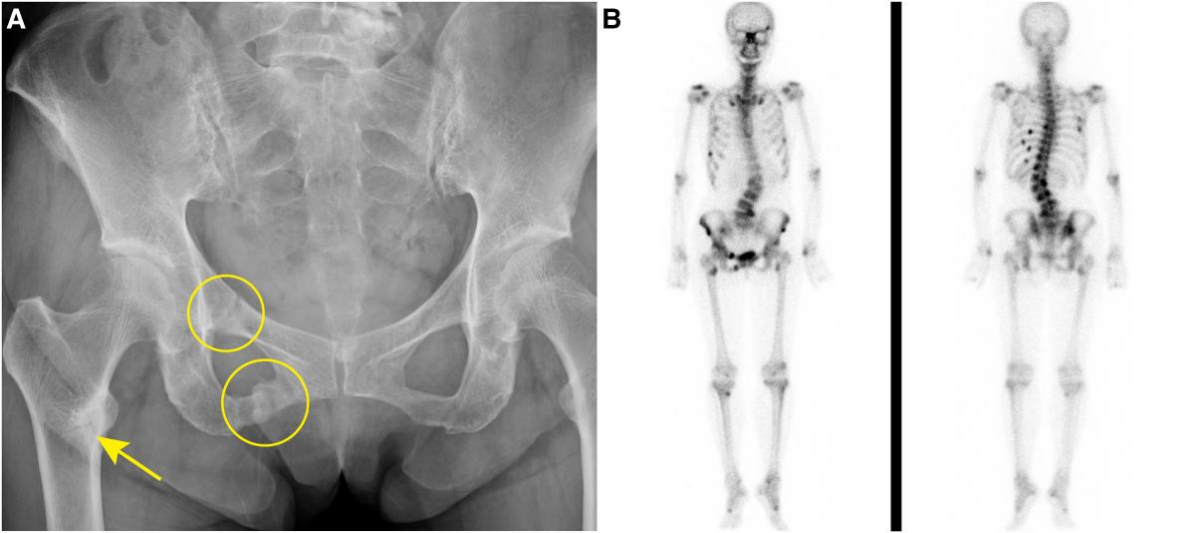
Pelvic radiography and bone scintigraphy. A, Pelvic radiography: pseudofracture of bones (Looser zone) are shown by yellow circles and a yellow arrow. B, Bone scintigraphy: There are multiple uptakes throughout the skeletal system.

Laboratory data showed elevated ALP levels of 255 U/L (reference range, 38-113 U/L) and decreased phosphate levels of 1.6 mg/dL (0.52 mmol/L). The percentage of tubular reabsorption of phosphate (%TRP) and the tubular maximum reabsorption of phosphate (TmP)/glomerular filtration rate (GFR) was decreased at 59.9% (reference range, 80%-94%) and 1.08 mg/dL (0.35 mmol/L) (reference range, 2.3-4.3 mg/dL; 0.74-1.39 mmol/L), respectively. Vitamin D deficiency was also suggested due to low serum 25(OH)D3 levels of 18.4 ng/mL (46 nmol/L) (reference range, 30-100 ng/mL; 75-250 nmol/L). Pelvic radiography showed Looser zone reflecting pseudofractures (Fig. 1A). Dual energy X-ray absorptiometry showed low left radial bone mineral density with 56% of the young adult mean. Whole-body bone scintigraphy revealed multiple fractures of the cranial bone, scapula, ribs, radial bone, spine, pelvis, and tibia (Fig. 1B).

**Figure 2.**
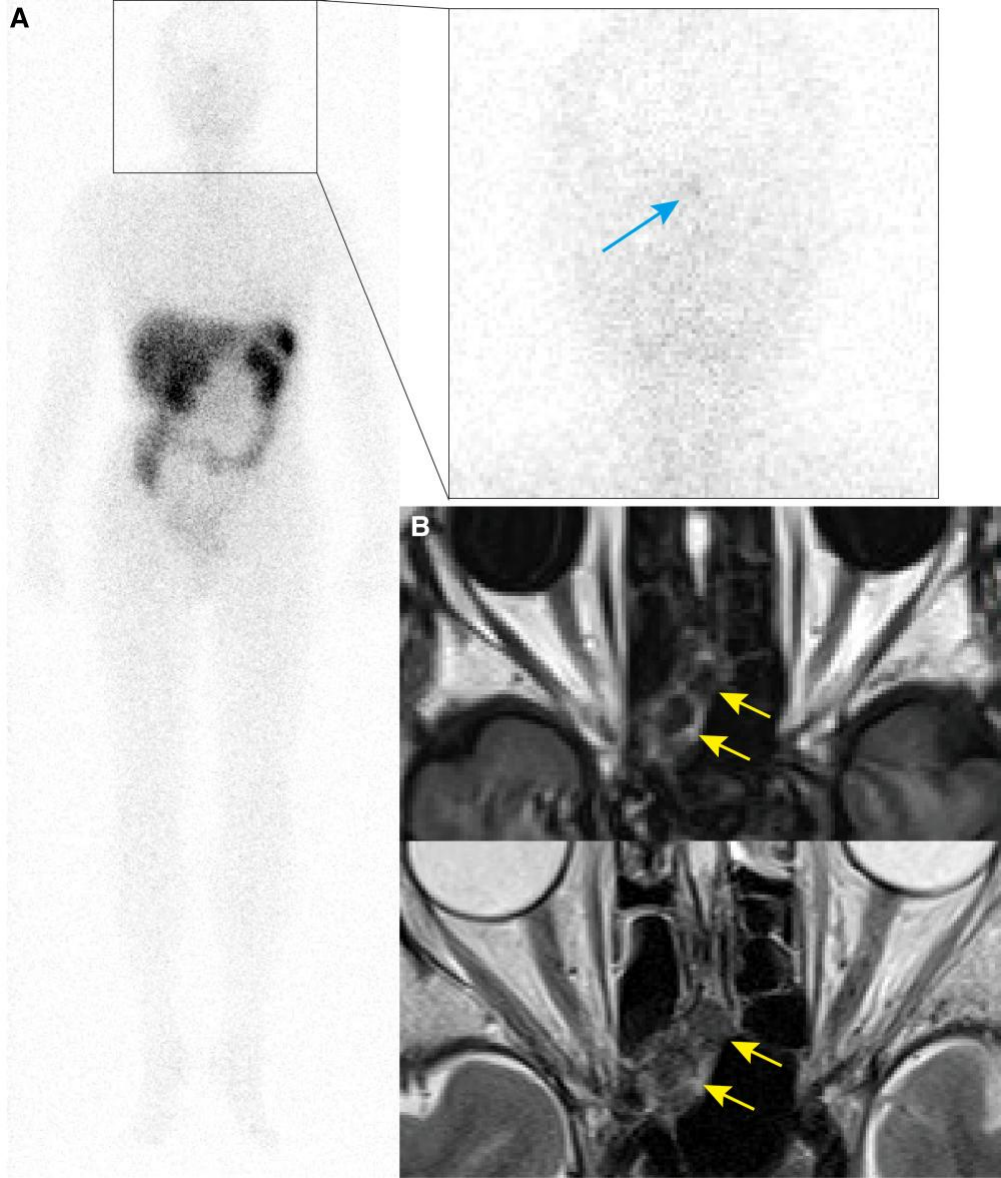
^111^In-pentetreotide scintigraphy and MR imaging. A, ^111^In-pentetreotide scintigraphy: A subtle uptake in the midline of the head (blue arrow). B, MR imaging: Mass-like lesions at posterior ethmoidal sinus with low signal intensity in both T1- and T2- weighted images (yellow arrows).

We diagnosed FGF23-related hypophosphatemic osteomalacia based on the following diagnostic criteria for osteomalacia [9]: hypophosphatemia, elevated serum ALP levels, symptoms of bone pain, low bone mineral density, multiple uptakes in bone scintigraphy, and radiography findings of Looser zone. ^111^In-pentetreotide scintigraphy performed to localize the tumor showed subtle uptake in the midline of the head (Fig. 2A). Magnetic resonance (MR) imaging of the head revealed a mass-like lesion at the posterior ethmoidal sinus with low signal intensity in both T1- and T2- weighted images. These findings were nonspecific but appeared to be inflammation or edema of the nasal mucosa (Fig. 2B). Physiological ^111^In-pentetreotide uptake in the pituitary gland was suspected but unconfirmed.

**Figure 3.**
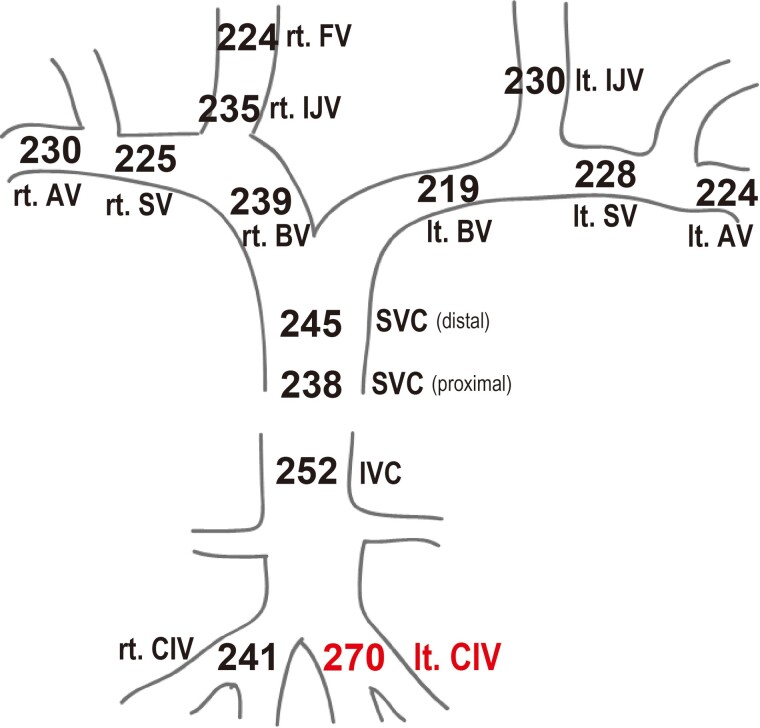
Systemic venous sampling of FGF23. Serum FGF23 levels (pg/mL) with the corresponding venous names; the highest being in the left common iliac vein, 270 pg/mL. Abbreviations: AV, axillary vein; BV, brachiocephalic vein; CIV, common iliac vein; FV, facial vein; IJV, internal jugular vein; IVC, inferior vena cava; lt., left; Rt., right; SV, subclavian vein; SVC, superior vena cava.

We therefore performed SVS of FGF23 to confirm the origin of FGF23 secretion. Surprisingly, serum FGF23 concentration was the highest in the left common iliac vein with 270 pg/mL (Fig. 3). This result suggested that the origin of FGF23 secretion was in the area around the pelvic cavity or left lower limb, which was in contrast with ^111^In-pentetreotide scintigraphy and MR imaging.

**Figure 4.**
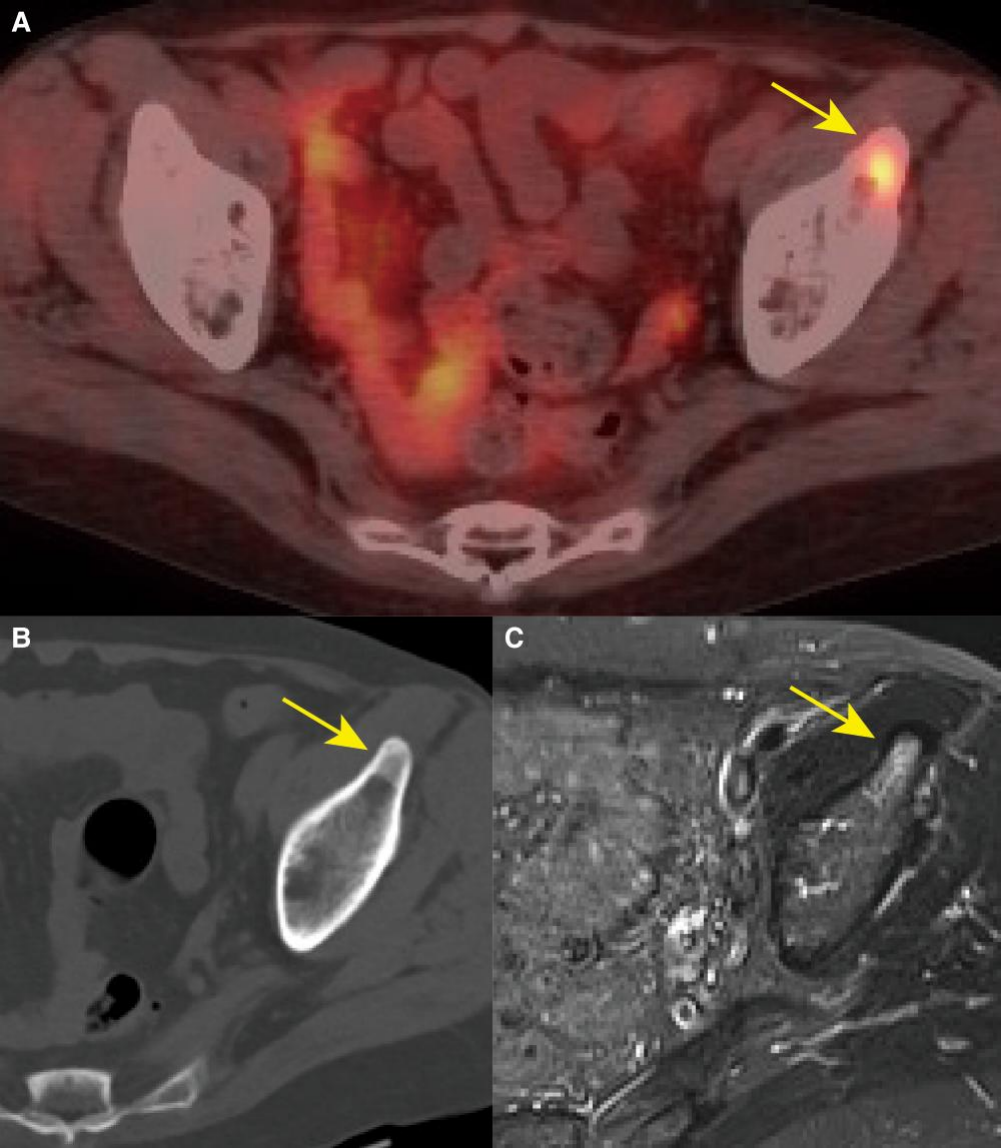
^68^Ga-DOTATOC PET/CT and pelvic CT and MR imaging. A, ^68^Ga-DOTATOC PET/CT: focal uptake in the left anterior inferior iliac spine (yellow arrow) clearly demonstrating the tumor location. B, CT imaging: A high-density area in the left anterior inferior iliac spine (yellow arrow). C, MR imaging: A high signal intensity of the left anterior inferior iliac spine on short tau inversion recovery image (yellow arrow).

Further evaluation with different modalities was required, so we consulted Kyoto University Hospital for ^68^Ga-DOTATOC PET/CT. The ^68^Ga-DOTATOC PET/CT clearly demonstrated focal uptake in the left anterior inferior iliac spine (Fig. 4A). Retrospectively analyzed, the subtle uptake detected by ^111^In-pentetreotide scintigraphy in the head was considered to be a physiological uptake in the pituitary gland. Although a high-density area in the CT scan and a high intensity area in MR images were shown in the left anterior inferior iliac spine (Fig. 4B and C), these findings were not specific enough to detect the location of tumor.

## Treatment

The patient started treatment with sodium phosphate 300 mg/day and alphacalcidol 1 µg/day at the first visit to our hospital. The dose of sodium phosphate was increased to 600 mg/day after 2 months (Table 1). Her bone pain gradually improved after supplementation of sodium phosphate and vitamin D. Based on the result of SVS and ^68^Ga-DOTATOC PET/CT, we decided that the culprit tumor was in the left anterior inferior iliac spine. The tumor was surgically resected.

**Table 1.** Perioperative laboratory data and treatment for tumor-induced osteomalacia

Laboratory data	Postoperative weeks						Reference range
	−29	−4	4	16	20	70	
Ca	9.0 mg/dL	9.1 mg/dL	9.0 mg/dL	9.2 mg/dL	9.0 mg/dL	9.6 mg/dL	8.8-10.1 mg/dL
	(2.24 mmol/L)	(2.27 mmol/L)	(2.25 mmol/L)	(2.30 mmol/L)	(2.25 mmol/L)	(2.40 mmol/L)	(2.20-2.52 mmol/L)
iP	1.6 mg/dL	2.9 mg/dL	2.8 mg/dL	2.1 mg/dL	3.2 mg/dL	4.2 mg/dL	2.7-4.6 mg/dL
	(0.52 mmol/L)	(0.94 mmol/L)	(0.90 mmol/L)	(0.68 mmol/L)	(1.03 mmol/L)	(1.36 mmol/L)	(0.87-1.49 mmol/L)
Cr	0.74 mg/dL	0.74 mg/dL	0.71 mg/dL	0.74 mg/dL	0.70 mg/dL	0.78 mg/dL	0.46-0.79 mg/dL
	(65.4 µmol/L)	(65.4 µmol/L)	(62.8 µmol/L)	(65.4 µmol/L)	(61.9 µmol/L)	(69.0 µmol/L)	(40.7-49.8 µmol/L)
%TRP	59.9%	50.7%	ND	75.3%	83.2%	88.6%	60-90%
ALP	255 U/L	264 U/L	211 U/L	280 U/L	233 U/L	110 U/L	38-113 U/L
intact PTH	69.1 pg/mL	ND	48.3 pg/mL	ND	ND	ND	9.3-74.9 pg/mL
	(7.32 pmol/L)		(5.12 pmol/L)				(0.99-7.94 pmol/L)
25(OH)VitD	18.4 ng/mL	ND	ND	ND	ND	ND	>30.0 ng/mL
	(46.0 nmol/L)						(>75.0 nmol/L)
FGF23	ND	226 pg/mL	74 pg/mL	108 pg/mL	ND	ND	<30 pg/mL
**Treatment**	**Dosage**						
Sodium phosphate	300 mg/day	600 mg/day	200 mg/day	0 mg/day	0 mg/day	0 mg/day	
Alfacalcidol	1.0 µg/day	1.0 µg/day	0.5 µg/day	0 µg/day	0 µg/day	0 µg/day	
Burosumab	0 mg/month	0 mg/month	0 mg/month	10 mg/month	20 mg/month	20 mg/month	

Abbreviations: ALP, alkaline phosphate; Ca, calcium; FGF23, fibroblast growth factor 23; iP, phosphate; ND, no data; (OH)VitD, hydroxy-vitamin D; PTH, parathyroid hormone; TRP, tubular reabsorption of phosphate.

## Outcome and Follow-Up

The pathological diagnosis was phosphaturic mesenchymal tumor. Hematoxylin and eosin staining showed that the tumor was composed of densely proliferated short spindle cells presenting an indistinct fascicular structure. Immunohistochemistry showed that the tumor cells were positive for SSTR2, and Ki67 index was lower than 1%. One month after surgery, the doses of sodium phosphate and alphacalcidol were reduced from 600 mg/day to 200 mg/day and 1.0 µg/day to 0.5 µg/day, respectively (Table 1). Four months after surgery, both sodium phosphate and alphacalcidol were stopped and %TRP was normalized; however, serum ALP levels (280 U/L) and serum FGF23 levels (108.0 pg/mL) remained high, indicating the presence of residual tumor (Table 1).

The patient declined our recommendation to undergo SVS or ^68^Ga-DOTATOC PET/CT again to search for residual tumors or another origin of FGF23 secretion. She instead opted for treatment with burosumab, a recombinant human anti-FGF23 monoclonal antibody. Four months after surgery, she started subcutaneous injection of burosumab 10 mg/month in place of sodium phosphate and alphacalcidol (Table 1). Serum phosphate and ALP levels improved within a month; from 2.1 mg/dL (0.68 mmol/L) to 3.2 mg/dL (1.03 mmol/L) and 280 U/L to 233 U/L, respectively. Her left foot pain gradually improved, and she could stand on her left leg after one month of treatment. Ten months after surgery, serum ALP levels remained high, so the burosumab was increased to 20 mg/month (Table 1). Eight months after this dose elevation, serum ALP levels had normalized to 110 U/L. Finally, she became free of bone pain and could walk without a cane and could ride a bicycle.

## Discussion

In the present case, we had difficulty in localizing the FGF23-producing tumor. Initially, we suspected the origin of FGF23 secretion to be the sinus lesion, based on ^111^In-pentetreotide uptake in the head and a mass-like lesion at posterior ethmoidal sinus detected by CT and MR imaging. However, SVS indicated the presence of a tumor around the pelvic cavity or left lower limb. According to these inconsistent results, we further evaluated the tumor location using ^68^Ga-DOTATOC PET/CT, which demonstrated a clear focal uptake in the left anterior inferior iliac spine, consistent with the location of the highest serum FGF23 levels in SVS. The culprit tumor in the left anterior inferior iliac spine was surgically resected and pathologically diagnosed as a phosphaturic mesenchymal tumor.

Careful interpretation of ^111^In-pentetreotide scintigraphy is required because of the physiological accumulation in normal organs including in the pituitary gland, thyroid, liver, kidneys, spleen, bladder, and bowel [7]. We could not construct fusion images between ^111^In-pentetreotide scintigraphy with CT, making it difficult to differentiate physiological accumulation in the pituitary gland and the sinus lesion.

The reported accuracy (sensitivity, specificity) varies, but ^68^Ga-DOTATOC PET/CT tends to have a higher sensitivity and specificity (60%-91%, 66%-95%) than those of ^111^In-pentetreotide scintigraphy (36%-95%, 64%) (Table 2).

**Table 2.** Comparison of the reported accuracy between ^111^In-pentetreotide scintigraphy and ^68^Ga-DOTATOC PET/CT

Modalities	Published paper [reference number]	Sensitivity	Specificity	Cases (n)
^68^Ga-DOTATOC PET/CT	Rendina et al [2]	91%	ND	274
	Hidaka et al [4]	87.5%	ND	9
	Paquet et al [10]	73%	66%	15
	Kato et al [11]	60%	95%	35
^111^In-pentetreotide scintigraphy	Rendina et al [2]	76%	ND	347
	Hidaka et al [4]	69.2%	ND	18
	El-Maouche et al [6]	36.3%	80.0%	11
	Kato et al [8]	72%	ND	18
	Chong et al [12]	95%	64%	31

Abbreviations: ND, no data; PET/CT, positron emission tomography/computed tomography.

A systematic review of 1725 TIO cases from Italy reported that ^68^Ga-DOTATOC PET/CT had a higher sensitivity than that of ^111^In-pentetreotide scintigraphy or ^18^F-FDG PET/CT [2]. Retrospective surveys for 88 Japanese patients with TIO also reported a higher sensitivity of ^68^Ga-DOTATOC PET/CT than ^111^In-pentetreotide scintigraphy; 87.5% and 69.2%, respectively [4].

Chong et al evaluated 31 subjects with TIO and reported that the specificity of ^111^In-pentetreotide was 64% [12]. Conversely, Kato et al analyzed 35 patients with TIO and showed that the specificity of ^68^Ga-DOTATOC PET/CT as high as 95% [11]; only one of their cases was determined to have false-positive uptake in a thyroid cyst.

Paquet et al evaluated the findings of scintigraphy in 15 patients with clinically diagnosed TIO; the sensitivity and specificity of ^68^Ga-DOTATOC PET/CT were 73% and 67%, respectively [10]. Notably, ^68^Ga-DOTATOC PET/CT could detect tumor locations in 5 out of 8 patients (63%) who had negative or a false-positive result of a previous ^111^In-pentetreotide scintigraphy [10].

^68^Ga-DOTATOC PET/CT has also been reported in relation to its efficacy for detecting small tumors causing TIO. Hidaka et al focused on FGF23-producing tumors < 10 mm; the detection rate of ^68^Ga-DOTATOC PET/CT (85.7%) was rather higher than that of ^111^In-pentetreotide (20%) [4]. In our patient, the tumor was < 10 mm in diameter, potentially explaining why the tumor was not detected by ^111^In-pentetreotide scintigraphy but detected by ^68^Ga-DOTATOC PET/CT.

SVS is another useful method for localizing FGF23-producing tumors that are not detected by CT or MR imaging [13]. A combination of SVS and ^68^Ga-DOTATOC PET/CT reportedly resulted in determining the precise localization of a tumor [8, 14]. In our present case, SVS contributed to excluding a physiological uptake in ^111^In-pentetreotide scintigraphy and led to the correct localization of the tumor by ^68^Ga-DOTATOC PET/CT.

A combination of SVS and ^68^Ga-DOTATOC PET/CT was useful in the present case for detecting a small-sized FGF23-producing tumor. Careful interpretation of scintigraphy is needed for precise localization of the tumor because the physiological uptake of radionuclides could lead to misdiagnosis.

## Learning Points

- ^68^Ga-DOTATOC PET/CT could visualize a ^111^In-pentetreotide-negative FGF23-producing tumor.
- The combination of ^68^Ga-DOTATOC PET/CT and systemic venous sampling for FGF23 was a valuable method for localizing an FGF23-producing tumor, especially because the tumor was small.
- FGF23-producing tumors often localize in the head or neck area, so physiologic uptake in the pituitary gland by SSTR scintigraphy should be carefully differentiated.

## Data Availability

Data sharing is not applicable to this article as no datasets were generated or analyzed during the current study.

## Abbreviations

ALP: alkaline phosphatase
CT: computed tomography
FGF23: fibroblast growth factor 23
MR: magnetic resonance
PET: positron emission tomography
SSTR: somatostatin receptor
SVS: systemic venous sampling
TIO: tumor-induced osteomalacia
TRP: tubular reabsorption of phosphate
